# The Role of Policy and Law in Shaping the Ethics and Quality of End-of-life Care in Intensive Care

**DOI:** 10.1007/s00134-022-06623-2

**Published:** 2022-01-22

**Authors:** Elizabeth Dzeng, Thomas Bein, J. Randall Curtis

**Affiliations:** 1. Division of Hospital Medicine, Department of Medicine, University of California, San Francisco, San Francisco, CA, USA; 2. Philip R. Lee Institute for Health Policy Studies, University of California, San Francisco, San Francisco, CA, USA; 3. Cicely Saunders Institute, King’s College London, London, England, UK; 4. University of Regensberg, Regensberg, Germany; 5. Cambia Palliative Care Center of Excellence at UW Medicine, University of Washington, Seattle, WA, USA; 6. Division of Pulmonary, Critical Care, and Sleep Medicine, Department of Medicine, University of Washington School of Medicine, Seattle, WA, USA

**Keywords:** institutional culture, palliative care, life-sustaining treatments, health policy, end-of-life care

The interplay between health policies and culture at the institutional, regional, and national level are important yet understudied questions in medicine. How do policies influence culture and vice versa? Does culture have more influence on policy, or does it more often flow the other direction? These are important empirical questions which have wide-ranging implications for both policy makers and those who want to improve clinical care. Institutional culture, or the collective values, beliefs, and behavioral norms of an institution, influences clinical practice patterns, behaviors, and attitudes [1]. Policies influence clinical practice patterns through direct influences of policy, but also indirectly through culture (Figure 1). It is thus essential to understand how the intended and unintended consequences of policy changes might positively or negatively impact clinician attitudes and behaviors, as well as patient and family outcomes. If employed strategically and thoughtfully institutional, regional, and national policy change can be a useful intervention to improve end-of-life care and foster institutional cultures that promote high-quality palliative care.

In this issue of *Intensive Care Medicine*, Lee and colleagues describe changes in clinicians’ perceptions of the quality of death in the intensive care unit following the implementation of South Korea’s Hospice, Palliative Care, and Life-sustaining Treatment Decision-Making Act (the “well-dying law”) in 2018, which legalized the ability for terminally ill patients to refuse CPR and life-sustaining treatments [2]. South Korea is a traditionally Confucian country, whose social norms are governed by strong familial relationships, filial piety, and a focus on collective harmony rather than individualism and autonomy [3]. Discussing death is seen as inappropriate and filial piety often interpreted as doing one’s best to ensure parents live as long as possible, even if treatments might not be beneficial [4, 5]. South Korea’s well-dying law appears to reflect the influence of shifting cultural norms on policy. As East Asian countries are increasingly exposed to Western attitudes and beliefs, notions of individual autonomy and self-determination become increasingly accepted [3].

The study by Lee and colleagues addresses the question of how policies influence institutional culture [2]. The authors found that clinicians’ assessment of quality of dying and death significantly improved after the passage of the law, as measured by the Quality of Dying and Death (QODD) questionnaire administered before (2016-2017) and after (2019-2020) the passage of the law. The authors found that since the law’s passage, fewer patients were admitted to the ICU for post-resuscitation care and the time from DNR to death was longer. Major limitations of the study include the inability to account for temporal changes other than the well-dying law and the lack of patient or family perspectives. Nonetheless, clinicians reported that discussions around end-of-life wishes, time to say goodbye to loved ones, maintenance of dignity and self-respect, and access to spiritual services all improved after the well-dying law was implemented.

The well-dying law is similar to other policies that have improved end-of-life care and acceptance of palliative care. These policies often increase the permissibility of limitations of life-sustaining treatment and provide clarity and transparency around the process through which these decisions are made. An example of this type of policy is France’s Leonetti law (2005), which focused on improving interprofessional communication about withdrawing or withholding of life-sustaining treatment during terminal illness [6]. Leonetti’s law created an explicit collegial interprofessional process for establishing consensus around limitations of non-beneficial life-sustaining treatment. In a qualitative study of French ICU clinicians following the passage of Leonetti’s Law, this interprofessional process was found to facilitate a more ethical climate around end-of-life decision-making, where discussions around treatment decisions shifted from hushed discussions to more openness and transparency [6]. Nurses felt more empowered to participate in end-of-life decision-making, thus reducing moral distress and clinicians felt they were better able to maintain a unified message with patients and families.

In contrast, laws which restrict options around withdrawing or withholding unwanted or non-beneficial life-sustaining treatments may have unintended consequences of constraining clinicians’ ability to act ethically. For example, Texas’s 2017 Senate Bill 11 (SB 11) enacted several restrictions around in-hospital do-not-resuscitate (DNR) orders, such as requiring written consent from a patient or surrogate to institute a DNR order or two witnesses in the event of a verbal consent [7]. Certain patient rights groups advocated for this law to discourage unilateral DNR orders, a rare practice believed to be frequent by these groups. The law had an immediate constraining effect on institutional culture. Some have noted that the law encourages a “culture of full code”, where risk-adverse physicians default to providing full resuscitation efforts regardless of whether resuscitation is in the patient’s best interest [8].

New York is another state whose laws evolved towards being more restrictive, constraining physicians’ ability to act in patients’ best interests. From 1988-2010, New York’s DNR law allowed a surrogate or two physicians to enter a DNR on a patient if resuscitation is deemed medically futile [9]. However, in 2010, New York passed the Family Health Care Decisions Act (FHCDA) which nullified the previous DNR law and subsumed DNR decision-making into a broader standard for the withdrawing and withholding life-sustaining treatments. Though technically the FHCDA’s standards for establishing futility were the same as the original DNR law, it was more difficult to apply and “forfeited the helpful specificity of the prior medical futility standards for DNR decisions” [9]. The law was less specific and prone to different interpretations and thus lacking in objective and quantitative measure. Importantly, the FHCDA forced physicians to burden surrogates with a false choice in circumstances where resuscitation was non-beneficial. This policy had a substantial effect on institutional culture at some New York hospitals where physicians felt obligated to provide non-beneficial resuscitation [1]. It encouraged a culture of unreflective deference to autonomy where physicians frequently felt uncomfortable making clinically-appropriate recommendations and unable to participate in shared decision-making. These changes created a culture rife with moral distress, particularly amongst medical trainees [10].

Policies regarding end-of-life care exist across a spectrum. Policies that restrict options have the potentially harmful effect of hindering ethically and clinically appropriate practices around limiting unwanted or non-beneficial life-sustaining treatment. On the other end of the spectrum, policies that provide the flexibility, clarity, and transparency to act ethically can improve the quality of end-of-life care and promote high-quality palliative care. More research is needed to understand the influence of and interaction between culture and policy at the institutional, regional, and national level to allow us to develop and support policies that improve the quality and ethics of care. Policy makers and clinicians must work together to create policies that promote ethical decision-making while refraining from overly restrictive policies that can be harmful to high-quality and ethical end-of-life care. The experiences in France [6] and South Korea [2] suggest that policy interventions have the power to change the culture around end-of-life care for the better.

## Figures and Tables

**Figure 1:**
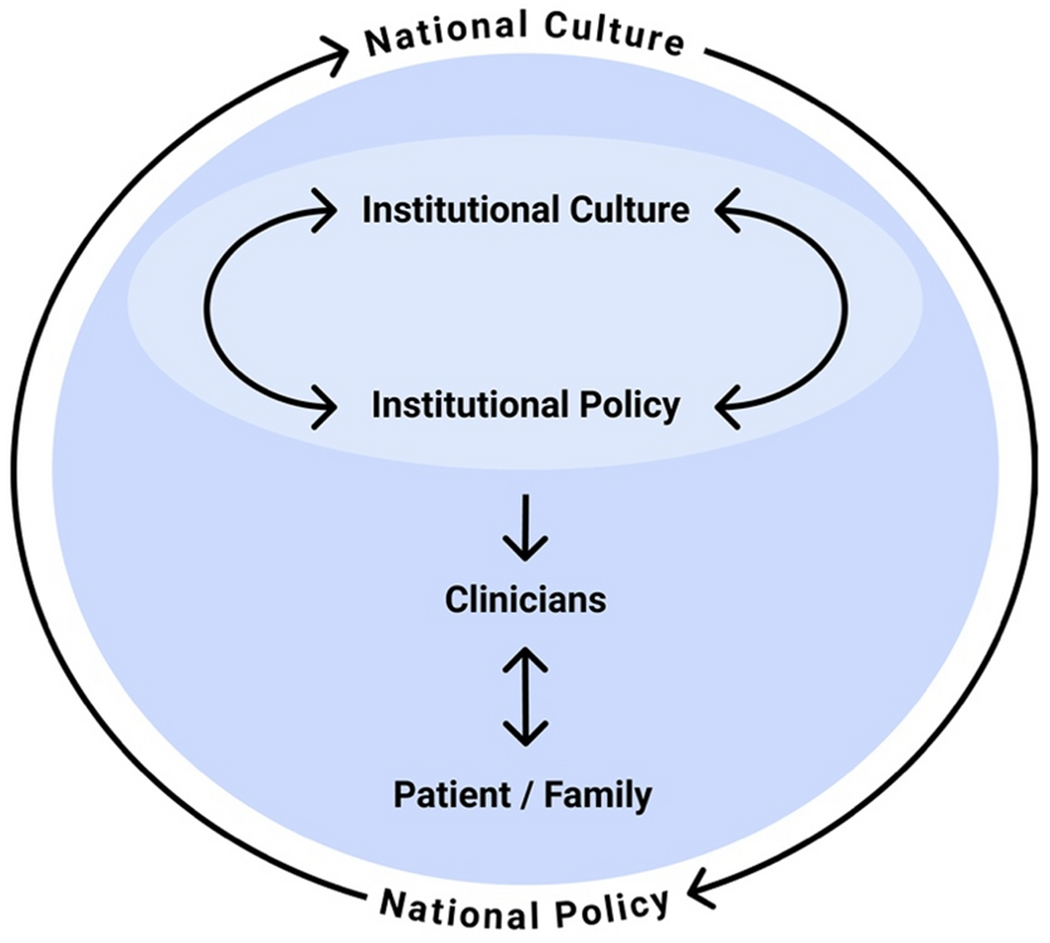
Conceptual framework for the influences of institutional and national cultures and policies on each other and on clinicians, patients, and families
